# Untargeted metabolomic analysis of metabolites related to body dysmorphic disorder (BDD)

**DOI:** 10.1007/s10142-023-00995-4

**Published:** 2023-02-28

**Authors:** Yawen Wang, Jinlong Huang

**Affiliations:** 1 Nanjing University of Chinese Medicine, No.138 Xianlin Road, Nanjing, 210023 Jiangsu China; 2 Department of Plastic Surgery, Affiliated Hospital of Nanjing University of Chinese Medicine, No.155, Hanzhong Road, Nanjing, 210000 Jiangsu China

**Keywords:** Body dysmorphic disorder, Metabolomics, Metabolites, Biosynthesis

## Abstract

Body dysmorphic disorder (BDD) is a disorder associated with depression and eating disorders. It often arises from minor defects in appearance or an individual imagining that he or she is defective. However, the mechanisms causing BDD remain unclear, and its pathogenesis and adjuvant treatment methods still need to be explored. Here, we employed a liquid chromatography-mass spectrometry (LC–MS)-based metabolomics approach to identify key metabolic differences in BDD versus healthy patients. We obtained plasma samples from two independent cohorts (including eight BDD patients and eight healthy control patients). Raw data were analyzed using Compound Discoverer to determine peak alignment, retention time correction, and extraction of peak areas. Metabolite structure identification was also obtained using Compound Discoverer by of accurate mass matching (< 10 ppm) and secondary spectral matching queries of compound databases. Next, multidimensional statistical analyses were performed using the *ropls* R package. These analyses included: unsupervised principal component analysis, supervised partial Least-Squares Discriminant Analysis, and orthogonal partial Least-Squares Discriminant Analysis. We then identified the most promising metabolic signatures associated with BDD across all metabolomic datasets. Principal component analysis showed changes in small-molecule metabolites in patients, and we also found significant differences in metabolite abundance between the BDD and normal groups. Our findings suggest that the occurrence of BDD may be related to metabolites participating in the following KEGG pathways: ABC transporters, purine metabolism, glycine, serine and threonine metabolism, pyrimidine, pyrimidine metabolism, biosynthesis of 12-, 14-, and 16-membered macrolides, microbial metabolism in diverse environments, biosynthesis of secondary metabolites, and caffeine and insect hormone biosynthesis.

## Introduction

Body dysmorphia disorder (BDD), also known as body image disturbance (BID), is a common mental and psychological problem in which an individual's body or appearance is objectively free from defects or has only slight defects, but is imagined by the individual is highly defective, thereby resulting in psychological pain. (Lacroix and Ranson 2021). Many studies have confirmed that social factors, including culture and economic status, have increased the attention people pay to their own body image, resulting in more cases of body image disorder (Akram et al. 2022). Female body image disorder has long been considered a common phenomenon, while male cases of body image disorder have been less well-studied (Day et al. 2022). However, many patients conceal their symptoms, so case prevalence statistics may underestimate how common it is (Moccia et al. 2022). The scientific literature has not come to a consensus on which specific therapy should be used to treat body image disorders, but drug therapy and psychological therapy are common (Graboyes et al. 2019). At present, the pathogenesis of body image disorder remains unclear, and appropriate methods of clinical treatment need to be further explored and improved.

Research has suggested that over time, more and more people have indicated that they feel troubled by their appearance, resulting in physical disorders that can lead to depression, serious illness, and even self-mutilation, and suicide (Brooks et al. 2020). Moreover, if medical staff lack awareness of BDD, BDD patients can easily become dissatisfied with medical treatments, which can trigger resistance, promote the development of psychological and physical problems, and increase the likelihood of altercations with medical staff and legal disputes (Demirdel and Ülger 2021). Appearance-induced depression is often accompanied by negative personality traits, including lower self-esteem, higher self-exclusion, and shame (Graboyes et al. 2020). Those individuals with high levels of depression often choose escapism in response to social demands, since social encounters prompt feelings of conversation anxiety and social avoidance (Pauzé et al. 2020).

Psychological counseling and drug treatments often accompany the clinical treatment of depression. The most common goal of drug treatment is to improve the cellular content and distribution of metabolites that exert anti-depressive effects (Stefanowski et al. 2017). Metabolites are downstream of gene expression and protein translation and metabolite abundance often depends on environmental factors; for this reason metabolites are thought to be closely related to individual phenotypes (Laíns et al. 2019). Metabolomics is a data-oriented research approach in which quantitative and qualitative data from the endogenous metabolome are obtained using high-throughput and high-sensitivity detection technologies (Roque and Romero 2021). After multivariate statistical analysis, the abundances of small (< 1 kDa) metabolites can be determined for different biological systems, including body fluids, cells, and tissues. Moreover, by combining metabolomic approaches with bioinformatics, key pathogenic metabolites and pathogenic metabolic pathways can be identified (Kumar et al. 2021). For example, a metabolomic analysis of the effect of methamphetamine administration on human monocyte-derived macrophages, identified eleven differential metabolites, including a new metabolite (m/z = 18) secreted by macrophages (Pawlak et al. 2019).

Untargeted stable isotope-resolved metabolomics is a recently developed framework for metabolomic analyses. It involves using a tracing isotope—such as 13C-labeled dietary fiber—to trace the metabolic fate of labeled molecules in different treatments. In one study, microbes extracted from rat feces were cultured in the presence of labeled fiber, and downstream analyses found that the use of inulin was significantly more efficient than the use of cellulose in the biosynthetic pathway, reflecting the fact that there are different metabolic pathways for dietary fiber in the gut microbiome. The use of this technique contributes to a deeper and more comprehensive understanding of the metabolic functions of the gut microbiome (Deng et al. 2021). In one study, a metabolomic approach was used to analyze plasma samples from of 15 healthy adolescents and 15 adolescents with PCOS (i.e., polycystic ovary syndrome). The results showed that PCOS may be related to abnormal lipid metabolism in the body. Another experiment showed that supplementation with two underexpressed metabolites was consistent with improving PCOS symptoms (Çelebier et al. 2020). In another study, a metabolomic analysis of urine from BALB/c mice infected with *Toxoplasma gondii* showed that *T. gondii* infection affected amino acid metabolism, fatty acid metabolism, and the nicotinate and nicotinamide metabolic pathways in mice; this information may be helpful for understanding the pathogenesis of toxoplasmosis and for improving treatment methods (Zhou et al. 2022).

Here, we used a non-targeted LC–MS-based metabolomic approach to study metabolite changes in BDD serum. Our aim was to identify the most relevant metabolic biomarkers and metabolic pathways that may help us understand and treat the biochemical impairment associated with this disease.

## Methods

### Participants

#### Disease group

Subjects were assessed using the Yale-Brown Obsessive–Compulsive Scale Modified for Body Dysmorphic Disorder (BDD-YBOCS) instrument. Patients with a score > 20 met the inclusion cutoff for the BDD group. In total, eight patients were selected for inclusion in the disease group.

#### Normal group

Subjects with a score > 20 as determined using the BDD-YBOCS. All subjects had no history of other psychiatric diseases, no clinical physical diseases, no history of drug abuse or allergy, no history of alcoholism, no special eating habits, and no abnormality in routine physical examination. Ages ranged from 20 to 75 years old, and we made no gender restrictions. In total, eight healthy adults were selected for inclusion in the normal group.

The study was approved by the Institutional Research Ethics Committee of the Affiliated Hospital of Nanjing University of Chinese Medicine. Each volunteer was given all basic information concerning the study and provided informed consent by signing the informed consent form.

### Collection of serum samples

All volunteers had venous blood drawn in the morning (at 8:00–10:00 am) on an empty stomach. Three milliliters of fresh venous blood was obtained from each patient through the middle cubital vein. Sampled blood was placed in a BD blood collection tube coated with a coagulant. The serum was separated by centrifugation at 3000 rpm/min for 15 min; then, 65 µL of the supernatant was dispensed into a 200 µL centrifuge tube. We recorded the patient ID on the tube and stored all tubes at − 80 ℃ until later use.

### LC–MS analysis

For LC–MS analysis, we first retrieved samples from the − 80 ℃ refrigerator and dissolved them on ice. Once liquid, we obtained 50 µL sample and added 1 mL extract solution (methanol:acetonitrile = 3:1, pre-cooled to − 4 ℃), vortexed for 5 min, sonicated for 15 min, then left to stand at 4 ℃ for 2 h. We then centrifuged all samples at 12,000 rpm at 4 ℃ for 15 min. The supernatant was then removed and concentrated to a dry powder by vacuum. The powder was then reconstituted by adding 100 µL of 50% methanol in water, and the resulting mixture was centrifuged at 2000 rpm at 4 ℃. Samples were vortexed for 3 min then re-centrifuged at 12,000 rpm at 4℃ for 15 min. The supernatant was then removed for injection analysis.

All extracted samples were placed in an 8 ℃ autosampler and separated using a HSS T3 column (Waters). The injection volume was 2 μL, the column temperature was 40 ℃, and the flow rate was 0.3 mL/min. The chromatographic mobile phase was as follows: A: 0.1% formic acid in water, B: 0.1% formic acid in methanol, C: 0.05% acetic acid in water, D: 0.05% acetic acid in methanol. The chromatographic gradient elution program was as follows: positive ion: 0–0.5 min, B: 2%; 0.5–6 min, B: 2%–50%; 6–10 min, B: 50%–98%; 10–14 min, B: 98%; 14–16 min, B: 98%–2%; 16–21 min, B: 2%. Negative ion: 0–0.5 min, D: 2%; 0.5–6 min, D: 2%–50%; 6–10 min, D: 50%–98%; 10–14 min, D: 98%; 14–16 min, D: 98%–2%; 16–21 min, D: 2%. QC standards were included in the samples to monitor system stability the reliability of the experimental data.

The samples were analyzed using electrospray ionization (ESI) in both positive and negative ion modes. The samples were separated by ultra-high performance liquid chromatography (UHPLC) using a Thermo Vanquish Liquid chromatograph then analyzed by mass spectrometry using a Thermo QE HF-X mass spectrometer. The conditions for mass spectrometry were as follows: ionization source: ESI ion source, sheath gas flow rate: 30; auxiliary gas: 10; spray voltage: 2.5 kV( +)/2.5 kV(-); S-Lens RF: 50; capillary temperature: 325℃; Gas temperature: 300 °C; secondary collision energy (NCE): 30; isolation window 1.5 m/z, Top N = 8. Scanning range: 70–1050 m/z, scanning mode: positive and negative ion scanning. Analyses of captured data was performed using Xcalibur version 4.1.

### Data preprocessing

Raw data were analyzed using Compound Discoverer (version 12,345) to identify peak alignment, retention time correction, and extraction of peak areas. Metabolite structure identification was also performed using Compound Discoverer by querying databases based on accurate mass matching (< 10 ppm) and secondary spectral matching search algorithms. Multidimensional statistical analysis—including principal component analysis (PCA), PLS-DA and OPLS-DA, was performed using the *ropls* package as implemented in the R statistical software (version 12 345).

## Results

### Experimental quality evaluation

**Fig. 1:**
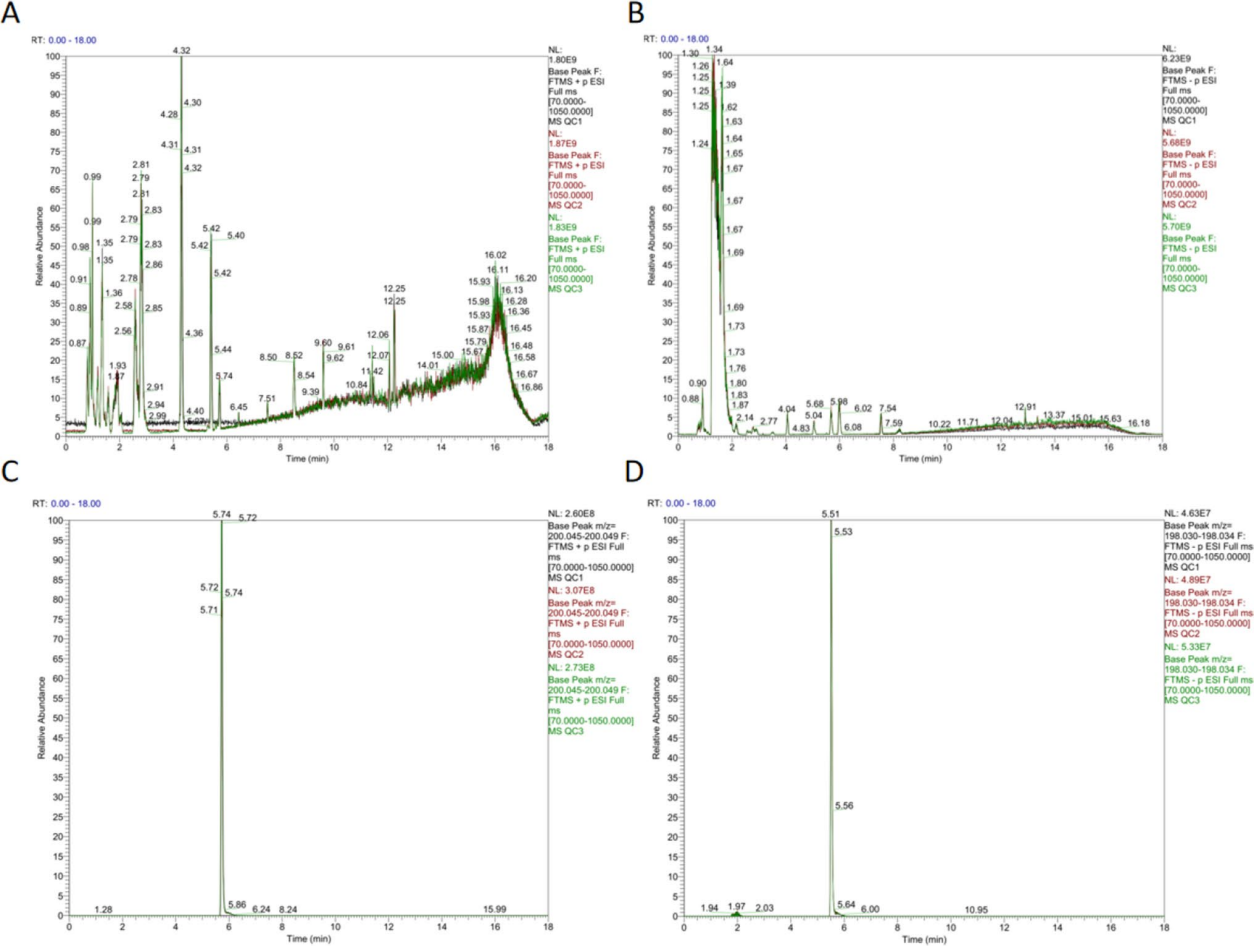
TIC and EIC maps of QC samples. **A** Pos TIC. **B** NEG TIC. **C** Pos EIC. **D** NEG EIC

We first evaluated and analyzed the system stability of our experiment by comparing the Total Ion Chromatography (TIC) map of QC samples and by analyzing PCA statistics of the overall sample. Total ion chromatograms from both the positive and negative ion detection modes of the mass spectrometer for the QC samples were compared. These results showed that the response intensity and retention time of each chromatographic peak overlapped, indicating that the instrumental error was small and the quality of data was reliable for this experiment (Fig. 1A and B). Next, by comparing the response peak height difference of the QC and blank samples, we assessed the stability of the detection process. We found that the retention times and response intensities of the QC samples were relatively stable, and we detected no obvious peaks of the internal standard in the blank samples. These results show that the data collection stability of the instrument is high, that very little material residue was found, and there was no cross-contamination between samples (Fig. 1C and D). As a whole, these results suggest that the LC–MS instrument and the detection process were accurate and reliable.

### PCA analysis of the metabolome

**Fig. 2:**
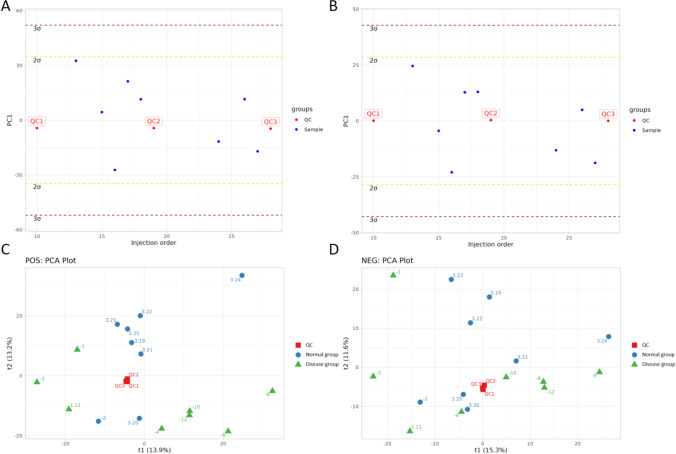
PCA score plots and one-dimensional PCA plots. **A** Pos PCA. **B** NEG PCA. **C** Pos One-dimensional PCA. **D** NEG One-dimensional PCA

PCA is an unsupervised data analysis method that involves selecting several comprehensive variables to dimensionally reduce a dataset containing different groups of samples. For this reason, here we examined metabolomic data collected in the previous step. In the resulting PCA one-dimensional distribution diagram (Fig. 2A and B), we found that the QC and samples were all within ± 2SD, indicating that our data was high quality and therefore suitable for further analyses. Next, we plotted the PC1 and PC2 dimensional maps and found that there was no apparent separation between samples for the POS (positive ion) and NEG (negative ion) mode data (Fig. 2C and D). Next, after sevenfold cross-validation we noted that the obtained PCA model parameters showed a BIDPRE (i.e., a type of principal component score) of 5 for POS, 0.523 cum for R2X, 5 for NEG, and 0.519 cum for R2X. Taken together, these results showed observable changes in small-molecule metabolite profiles among patients.

### PLS-DA and OPLS-DA analyses of the metabolome

**Fig. 3:**
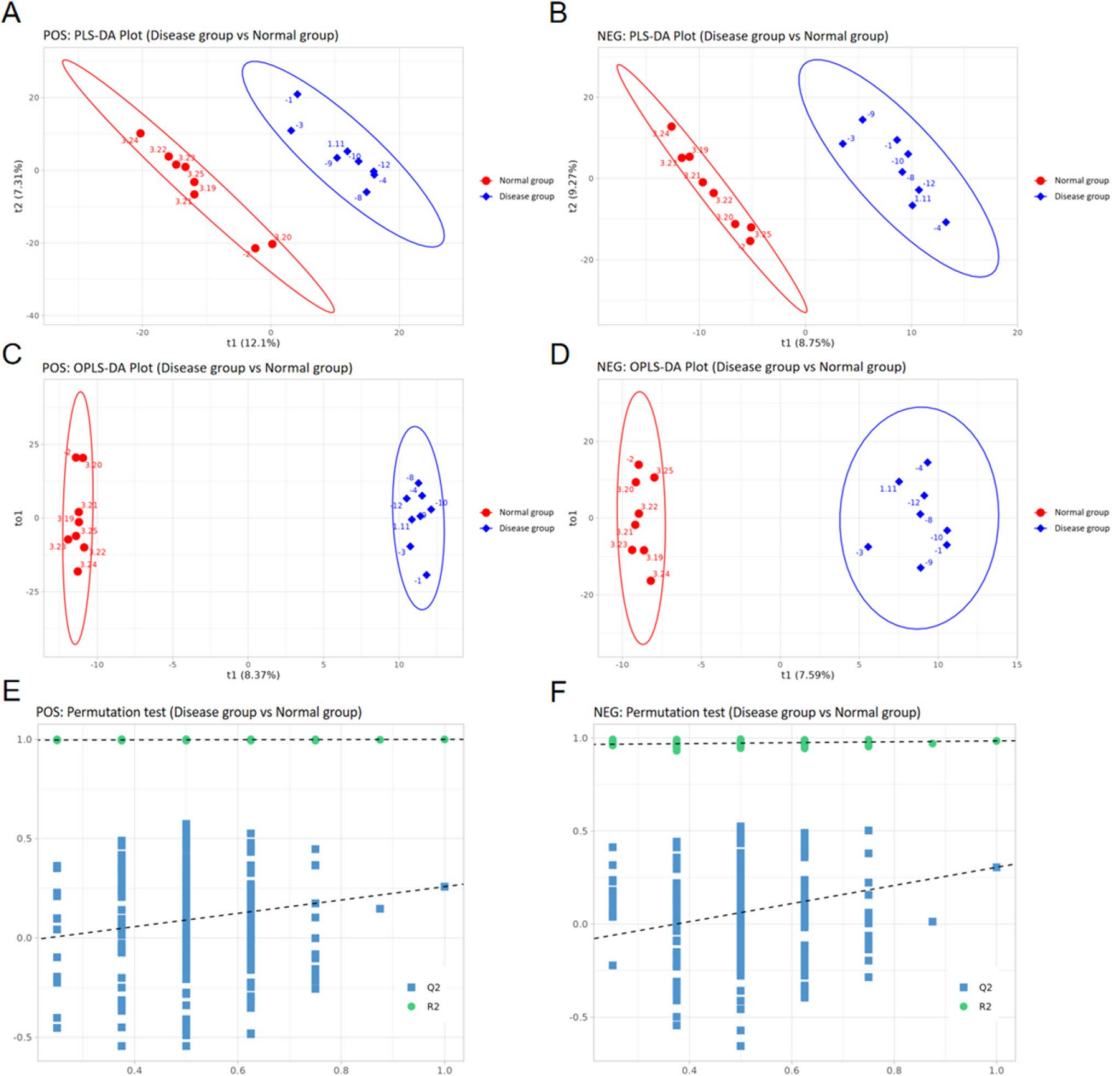
PLS-DA and OPLS-DA results. **A** Pos PLS-DA. **B** NEG PLS-DA. **C** Pos OPLS-DA. **D** NEG OPLS-DA

Partial Least-Squares Discriminant Analysis (PLS-DA) is a supervised data analysis method that uses partial least-squares regression to estimate a model between a number of data points (e.g., metabolites) and one of more categories. This model can then be used to predict the category of the sample. A statistically drawn PLS-DA score map (Fig. 3A and B) shows that our model has a high degree of interpretability for the Y-axis variable dataset. Orthogonal Projections to Latent Structures Discriminant Analysis (OPLS-DA) is another supervised data analysis method that is a refinement of PLS-DA, since it filters out noise irrelevant to classification information, and thereby improves the analytical ability and effectiveness of the model. A statistically drawn OPLS-DA score chart shows that the intra-group difference for both the disease and normal groups is small, while the inter-group difference is large (Fig. 3C and D). By analyzing OPLS-DA model scores for the two groups (Fig. 3E and F), we found that the OPLS-DA model did not overfit data from the POS and NEG datasets.

### Screening for differential metabolite profiles

**Fig. 4:**
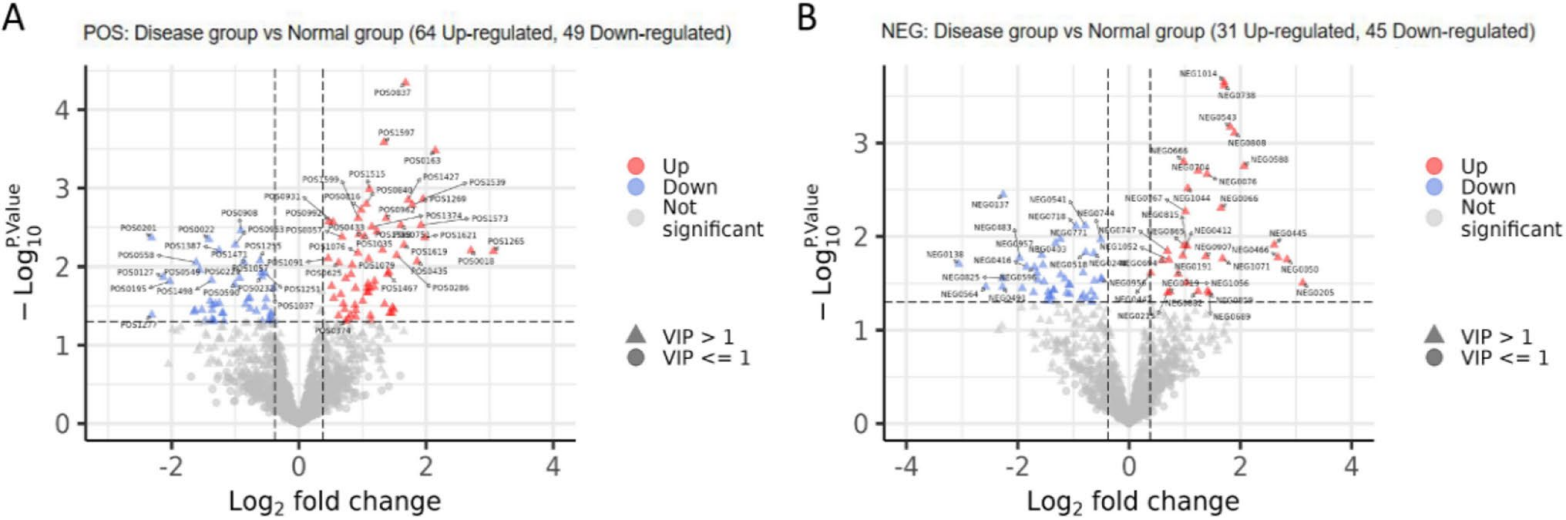
Volcano plots of differentially expressed metabolites. **A** Pos. **B** NEG

Next, we used univariate statistical analyses, including a linear statistical model and a Bayesian algorithm (implemented in the limma package in R) to analyze the obtained data. Using these analyses, we obtained a mean normalized quantitative value (AveExpr), fold change (FC), t value (i.e., *T*-test score), *P*-value, and adjusted *P-*value (Adj.P.Val) for the differential metabolite profiles of the two groups. In this experiment, we used fold difference |FC|≥ 1.3 and *P* < 0.05 as screening criteria for identifying differentially expressed or differentially abundant metabolites. Using these criteria, we found 64 metabolites that were upregulated and 49 metabolites that were downregulated using the positive ion dataset, as well as 31 metabolites that were upregulated and 45 that were downregulated using the negative ion dataset (Fig. 4A and B).

### Bioinformatic analyses

**Fig. 5:**
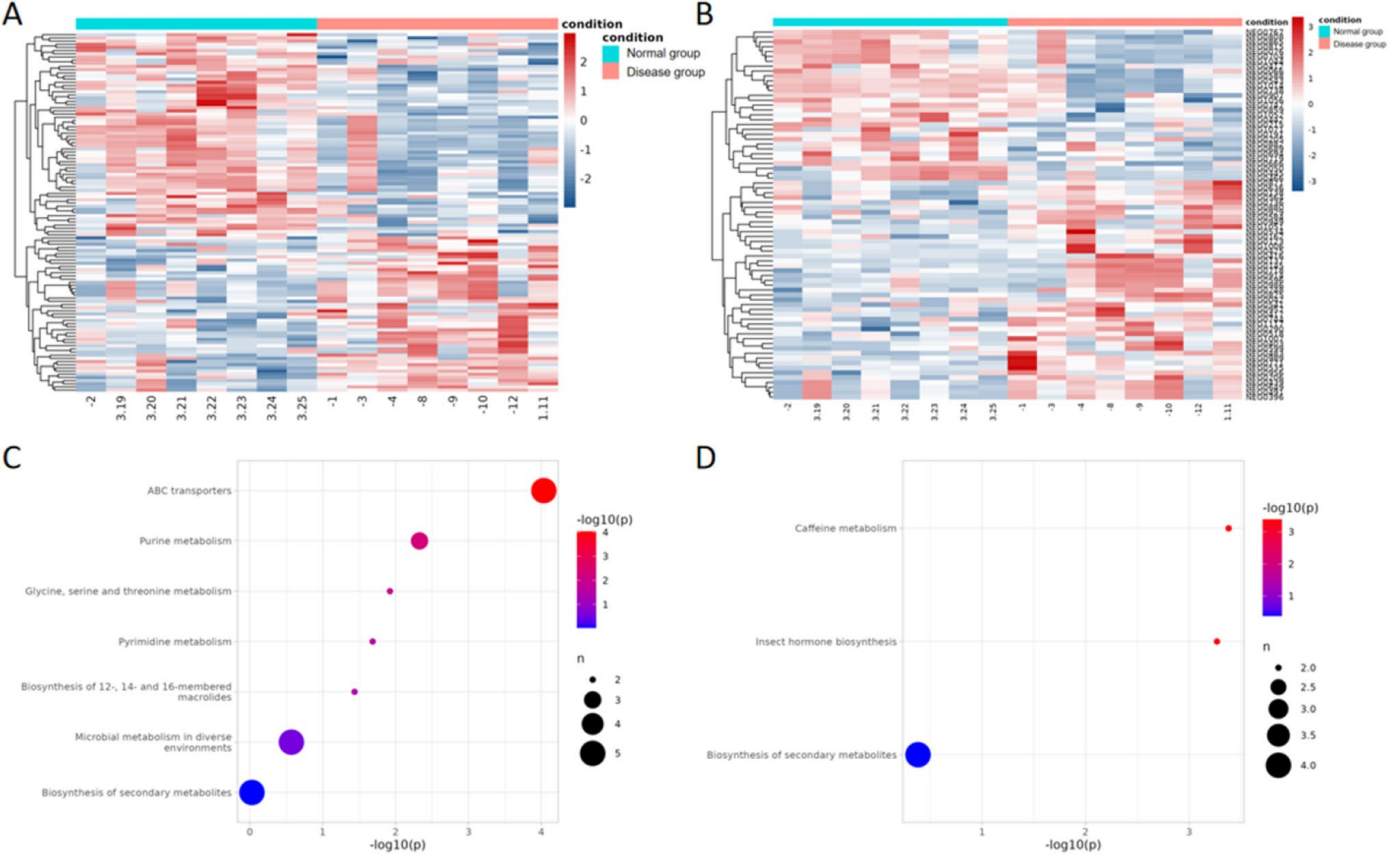
Hierarchical clustering analysis of differentially expressed metabolites and KEGG metabolic pathway analysis. **A** Pos hierarchical clustering results. **B** NEG hierarchical clustering results. **C** Pos KEGG analysis. **D** NEG KEGG analysis

To evaluate the functions of differentially expressed metabolites, and to more comprehensively and intuitively display the relationships between samples and differences in metabolite expression associated with BDD, we performed a hierarchical clustering analysis on the differentially expressed metabolites identified above. This analysis can help us to accurately screen for marker metabolites related to BDD and to determine which metabolic processes are related to BDD (Fig. 5A and B). We first annotated all differential expressed metabolites by KEGG ID using Thermo Scientific Compound Discoverer. We then queried the KEGG metabolic pathway database to identify which pathways were enriched in differentially expressed metabolites (Fig. 5C and D). Among them, the POS dataset was found to contain metabolites related to ABC transporters; purine metabolism; glycine, serine and threonine metabolism; pyrimidine metabolism; biosynthesis of 12-, 14-, and 16-membered macrolides; microbial metabolism in diverse environments; and biosynthesis of secondary metabolites. Metabolites in the NEG dataset were mainly related to caffeine metabolism; insect hormone biosynthesis; and biosynthesis of secondary metabolites. These results, which include the identification of differentially expressed metabolites and the KEGG pathways enriched in differentially expressed metabolites, provide useful information for further study of the mechanisms associated with depression in patients with BDD.

## Discussion

Patients with BDD consider their appearance to be unattractive or ugly. This dissatisfaction may include any or all parts of the body, including the appearance of their face (Akram et al. 2022). The diagnosis of BDD relies on clinical and scale diagnoses, mainly based on clinical observation and on the DSM-IVh or CCMD-3. Due to issues such as patient judgment and private privacy, scale diagnosis is mainly used as a supplemental diagnosis (Häberle et al. 2020). BDD patients are also often affected by mental disorders. While the progression of BDD can begin with slight body image distortion, it can develop to the point where it produces anxiety and depression (Hamamoto et al. 2019). Next, in response to anxiety and depression patients may feel the need to escape from reality, abuse drugs, drink alcohol, self-abuse, engage in self-inflicted surgery, or even to commit suicide (Gupta and Kapur 2019). Depression is only one of the comorbidities of BDD, but it is generally the most serious (Sobanski and Schmidt 2000). Due to the lack of qualified psychiatrists in the plastic surgery department, these facilities generally screen patients only through scales. BDD-YBOCS was translated and revised by our team with good reliability and validity (Chen et al. 2022).

Depression is a persistent and serious mental illness that affects more than 120 million people worldwide (Peng et al. 2015). In the USA, more than 19 million adults are reported to suffer from depression (Baglioni et al. 2011), while more than 26 million people in China do as well (He and Zhang 2013). Depression is now the most important risk factor for suicide, with a population-attributable risk ratio of approximately 28% (Miret et al. 2013), which is projected by 2030 to be the leading cause of death worldwide (Tian et al. 2013).

Using a proton NMR-based metabolomics protocol, one study identified 23 differentially expressed metabolites between subjects with major depressive disorder and healthy control subjects. Moreover, five of these metabolites were singled out as potential markers of depression (Zheng et al. 2013). Furthermore, in a plasma metabolomic analysis of chronic unpredicted mild stress in a rat depression model, another recent study found that glucose metabolism is related to depression (Chen et al. 2015). Despite extensive research into the mechanisms underlying depression, its causes and the development of effective treatments still remain poorly understood. In this paper, we conducted a metabolomic analysis of the serum of patients with depression caused by BDD, and this information has identified potential biomarkers that may be used for further study of the mechanisms responsible for depression caused by BDD.

The potential biomarkers we identify in this study have different functions. ABC transporters, also known as efflux pumps, are powered by ATP hydrolysis and mediate the transmembrane transport of various endogenous and exogenous molecules (Hartz and Bauer 2011). In the brain, these transporters clear toxic peptides (Jha et al. 2019) and compounds to maintain normal brain homeostasis; they are also associated with multidrug resistance (MDR) brain cancers (Wang et al. 2021). Inborn errors of purine metabolism are known to cause neurological pediatric syndromes (Camici et al. 2010) and chronic mountain sickness (Gao et al. 2020). Glycine, serine, and threonine metabolism are associated with male infertility (Ma et al. 2019) and diabetic kidney disease (Hyeon et al. 2020). Pyrimidine metabolism is associated with poor prognosis in liver cancer (Wang et al. 2020) and mitochondrial damage (Micheli and Sestini 2011). There are few reports regarding the biosynthesis of 12-, 14-, and 16-membered macrolides, and these mainly focus on antibiotics (Rubinstein 2001). Finally, caffeine metabolism is associated with sleep and anxiety, and is a site of potential neurodegenerative and psychiatric disorders (Nehlig 2018).

At present, metabonomic analysis mainly relies on several key technologies, including: nuclear magnetic resonance spectrometry (NMR), gas phase chromatography (GC), liquid phase chromatography (LC), capillary electrophoresis (CE), and mass spectrometry. However, none of these technologies can comprehensively identify and quantify the whole metabolome from a biological sample, and each technology has both advantages and disadvantages (Chacko et al. 2022 & Trifonova et al. 2021). At present, MS is the main detection method. However, one of its limitations is that it can only be used to identify known or standard metabolites and is not suitable for identifying new ones. In contrast, NMR does not require knowledge of metabolite characteristics in advance, but can only perform semi quantitative analyses. Even without proper annotation, NMR can identify significant peaks, thereby adding critical complexity to biochemical and mechanistic characterizations of metabolites. GC can be used with purpose-built columns to separate out specific analytes of interest, but the analytes themselves need to be stable at high temperatures. With respect to MS, the quantity of substances detected by LC/MS is greater than GC/MS, but the accuracy and quantification can be poor, and it is difficult to detect polar and ionic compounds (Yao et al. 2019). Recently, UHPLC has been developed to improve peak resolution, speed, and sensitivity (Plumb et al. 2022). When dealing with highly polar and/or charged metabolites CE is a common first choice, but it has low flux and can be expensive, so it is generally used for the analysis of specific metabolites (Stolz et al. 2019). Here, we used a combination of UHPLC and MS to identify differentially expressed metabolites, but the nature of these methods means that our capacity to detect highly polar metabolites or those with highly charged ions is relatively weak. Thus, future work using additional experimental approaches may be beneficial to further supplement our data.

## Conclusions

In conclusion, we collected plasma samples from eight BDD patients and eight healthy control subjects. We then used an LC–MS metabolomic framework to identify metabolic differences between the control and BDD experimental groups. We identified metabolic differences in the biosynthesis of secondary metabolites, microbial metabolism in diverse environments, and ABC transporters; these may be related to key metabolic events in BDD. Moreover, the accumulation of purine, pyrimidine, macrolides, and secondary metabolites suggests an important role for substance metabolism in nerves. These data may improve our understanding of the pathogenesis of BDD and facilitate disease detection and intervention.

## Data Availability

All the data during the current study are included in the article.
